# Data analysis of PD-1 antibody in the treatment of melanoma patients

**DOI:** 10.1016/j.dib.2020.105523

**Published:** 2020-04-12

**Authors:** Ting Li, Chao Zhang, Gang Zhao, Xinwei Zhang, Mengze Hao, Shafat Hassan, Min Zhang, Hong Zheng, Da Yang, Liang Liu, Farideh Mehraein-Ghomi, Xu Bai, Kexin Chen, Wei Zhang, Jilong Yang

**Affiliations:** a Department of Bone and Soft Tissue Tumor, Tianjin Medical University Cancer Institute and Hospital, Tianjin 300060, China; b National Clinical Research Center for Cancer, Key Laboratory of Cancer Prevention and Therapy, Tianjin's Clinical Research Center for Cancer, Key Laboratory of Molecular Cancer Epidemiology, Tianjin, China; c Department of Pathology, Tianjin Medical University Cancer Institute and Hospital, Tianjin 300060, China; d Departments of Biotherapy, Tianjin Medical University Cancer Institute and Hospital, Tianjin 300060, China; e Department of Stem Cell Transplantation, Institute of Hematology and Blood Diseases Hospital, Chinese Academy of Medical Science & Peking Union Medical College, Tianjin 300060, China; f Department of Pharmaceutical Sciences, University of Pittsburgh, Pittsburgh, PA 15261, United States; g Department of Epidemiology and Biostatistics, Tianjin Medical University Cancer Institute and Hospital, Tianjin 300060, China; h Center for Cancer Genomics and Precision Oncology, Wake Forest Baptist Comprehensive Cancer Center, Winston-Salem, NC 27157, United States; i Department of Cancer Biology, Wake Forest School of Medicine, Winston-Salem, NC 27157, United States; j Department of Radiology, Tianjin Medical University Cancer Institute and Hospital, Tianjin 300060, China

**Keywords:** Melanoma, Pd-1/PD-L1, IGFBP2, Immunotherapy

## Abstract

Data presented in this article are supplementary materials to the research article entitled “IGFBP2 regulates PD-L1 expression by activating the EGFR-STAT3 signaling pathway in malignant melanoma”. Data for melanoma patients who did not receive anti-PD-1 treatment were obtained from Tianjin Medical University Cancer Institute & Hospital from February 1981 to May 2013. Kaplan–Meier was used for survival analysis. RNA sequencing data from 28 melanoma patients receiving anti-PD-1 therapy were download from GEO database (GSE78220). Cluster analysis of RNA expression was performed using R (package pheatmap). The difference of PD-L1 expression was analysed by the Boxplot (R ggplot2 package). Differences between each group were analyzed by Fisher exact test. Information of 13 melanoma patients who had failed prior chemotherapy and treated in the Tianjin Medical University Cancer Institute & Hospital between July 2015 and December 2018 was collected. The response was captured by Response Evaluation Criteria in Solid Tumors 1.1 (RECIST 1.1).

Specifications table

**Table:** 

Subject	Cancer Research
Specific subject area	Malignant melanoma, anti-PD-1, biomarker
Type of data	Table and Figure
How data were acquired	Download from GEO database, Microscope and survey
Data format	Raw Analyzed
Parameters for data collection	The downloaded raw data is standardized before data analysis. Microscope photos should be of sufficient resolution.
Description of data collection	Data for melanoma patients who did not receive anti-PD-1 therapy were obtained from Tianjin Medical University Cancer Institute & Hospital from February 1981 to May 2013. RNA sequencing data from 28 melanoma patients receiving anti-PD-1 therapy were download from the GEO database (GSE78220). Information of 13 melanoma patients who had failed prior chemotherapy and treated in the Tianjin Medical University Cancer Institute & Hospital between July 2015 and December 2018 was collected.
Data source location	Department of Bone and Soft Tissue Tumor, Tianjin Medical University Cancer Hospital & Institute, Tianjin 300,060, China
Data accessibility	With the article
Related research article	Ting, L. et al. IGFBP2 regulates PD-L1 expression by activating the EGFR-STAT3 signaling pathway in malignant melanoma. Cancer Letters, 2020, 477(2020):19–30.

## Value of the data

• The present data show the characteristics of melanoma patients with or without anti-PD-1 treatment from RNA and protein levels. The data might contain valuable information on the clinical use of anti-PD-1 agents.
• These data are preliminary exploration of combined IGFBP2 and PD-L1 as reliable biomarkers to predict the efficacy of anti-PD-1/PD-L1 therapy. These data and methods provide direction for further expanding research and other reliable biomarkers.

## 1 Data description

**Fig. 1:**
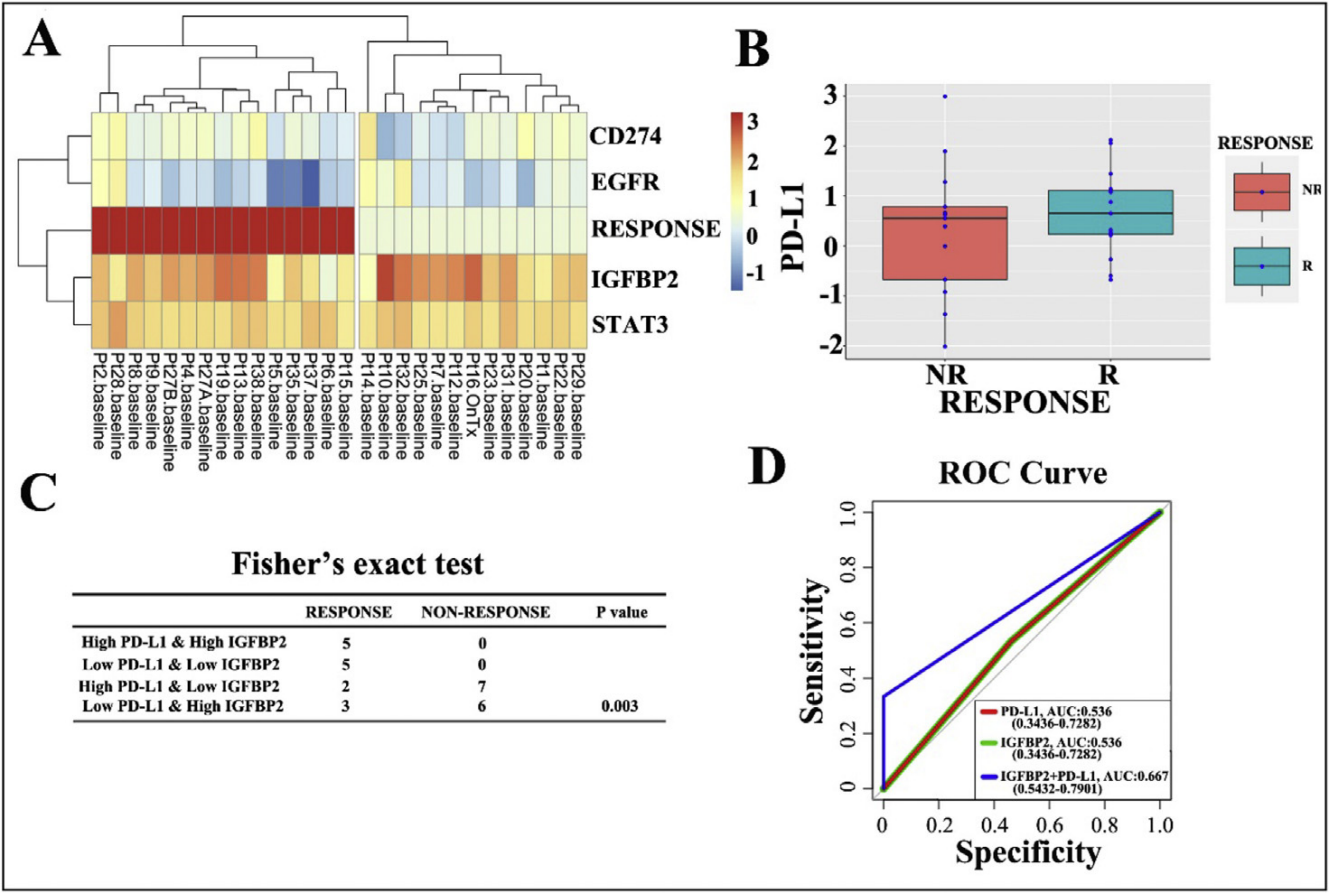
The mRNA expression levels of IGFBP2 and PD-L1 and the predict role of anti-PD-1 treatment. (A) The expression levels of PD-L1-related genes, including PD-L1 (CD274) itself, in 28 melanoma patients (GSE78220). The mRNA expression levels of genes with hypothetical roles in modulating response to anti-PD-1 therapy from RNA-seq data GSE78220 of 28 melanoma patients who received anti-PD-1 treatment. RESPONSE: response to anti-PD-1 treatment. The red box indicates a positive response. (B) Differential expression of PD-L1 in mRNA levels in melanoma from responders (R) and non-responders (NR). (C) Fisher's exact test were used to indicates the efficiency of anti-PD-1treatment on different groups of melanoma patients. (*p* < 0.05). (D) ROC curve of shows the AUC of both high PD-L1 and high IGFBP2 mRNA group, high IGFBP2 mRNA expression group and high PD-L1 mRNA expression group (AUC: 0.667 vs. 0.536 vs. 0.536).

**Fig. 2:**
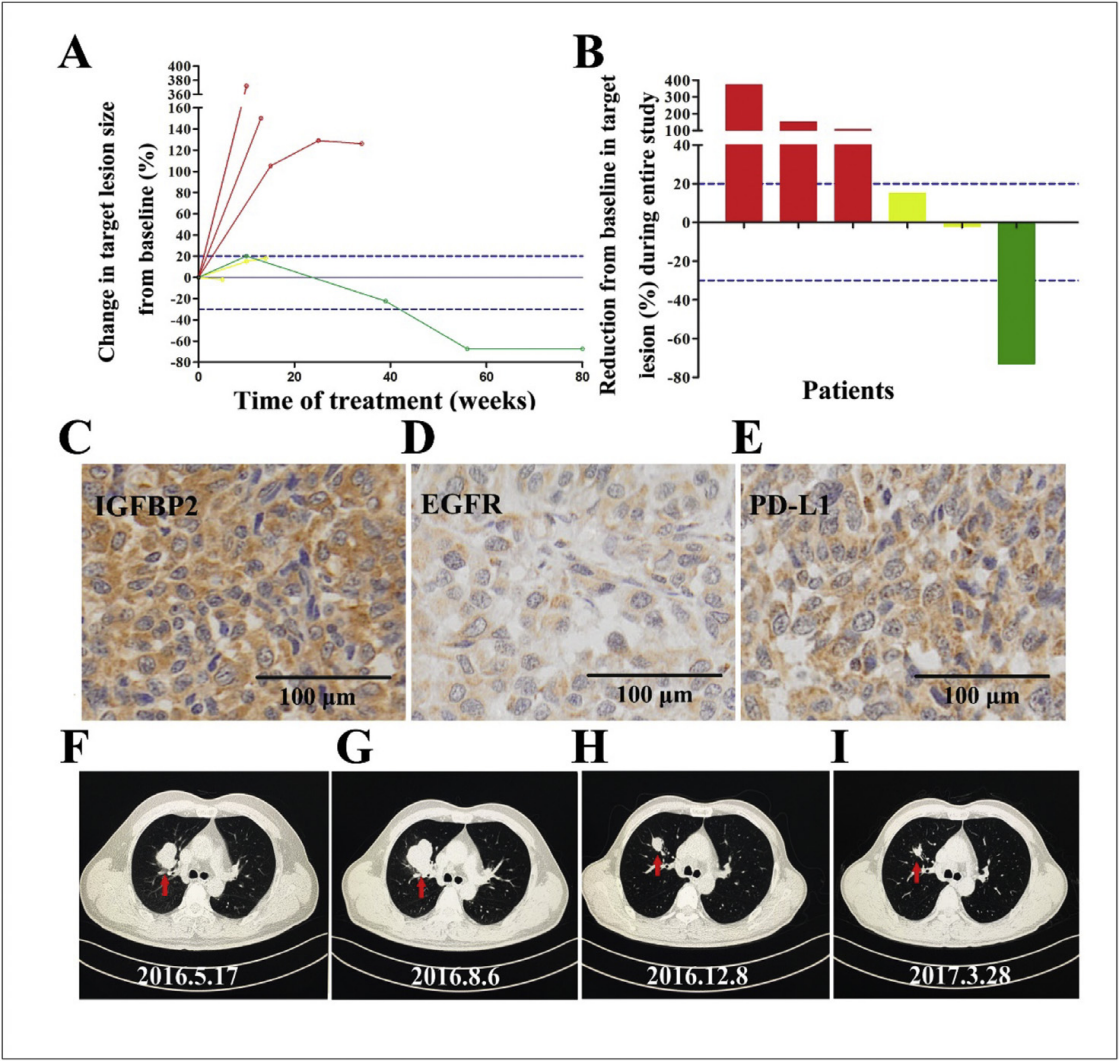
The efficacy of anti-PD-1 treatment and the expression of IGFBP2, EGFR and PD-L1. (A) Changes in the size of the target lesions after anti-PD-1 treatment compared with the baseline in 6 melanoma patients with measurable lesions. The green line shows that the target lesions shrank more than 30% by the final measurement. The red lines show that the target lesions increased by 20% by the final measurement. The yellow lines represent the target lesions that changed between 20% and −30%. One patient achieved PR, two patients achieved SD and three patients suffered from PD. (B) The maximum change in the target lesions in 6 melanoma patients treated with Keytruda or Opdivo was evaluated by RECIST 1.1. (C, D, E) The pathological data of one patient with lung metastatic melanoma and response to anti-PD-1 treatment showed high IGFBP2 (C) EGFR (D) and PD-L1 (E) expression. (F, G, H, I) The repeated chest CT showed the PR patient with increased volumes of lung metastases at 2.6 months (F-G) and then a gradual decrease (H-I).

**Fig. 3:**
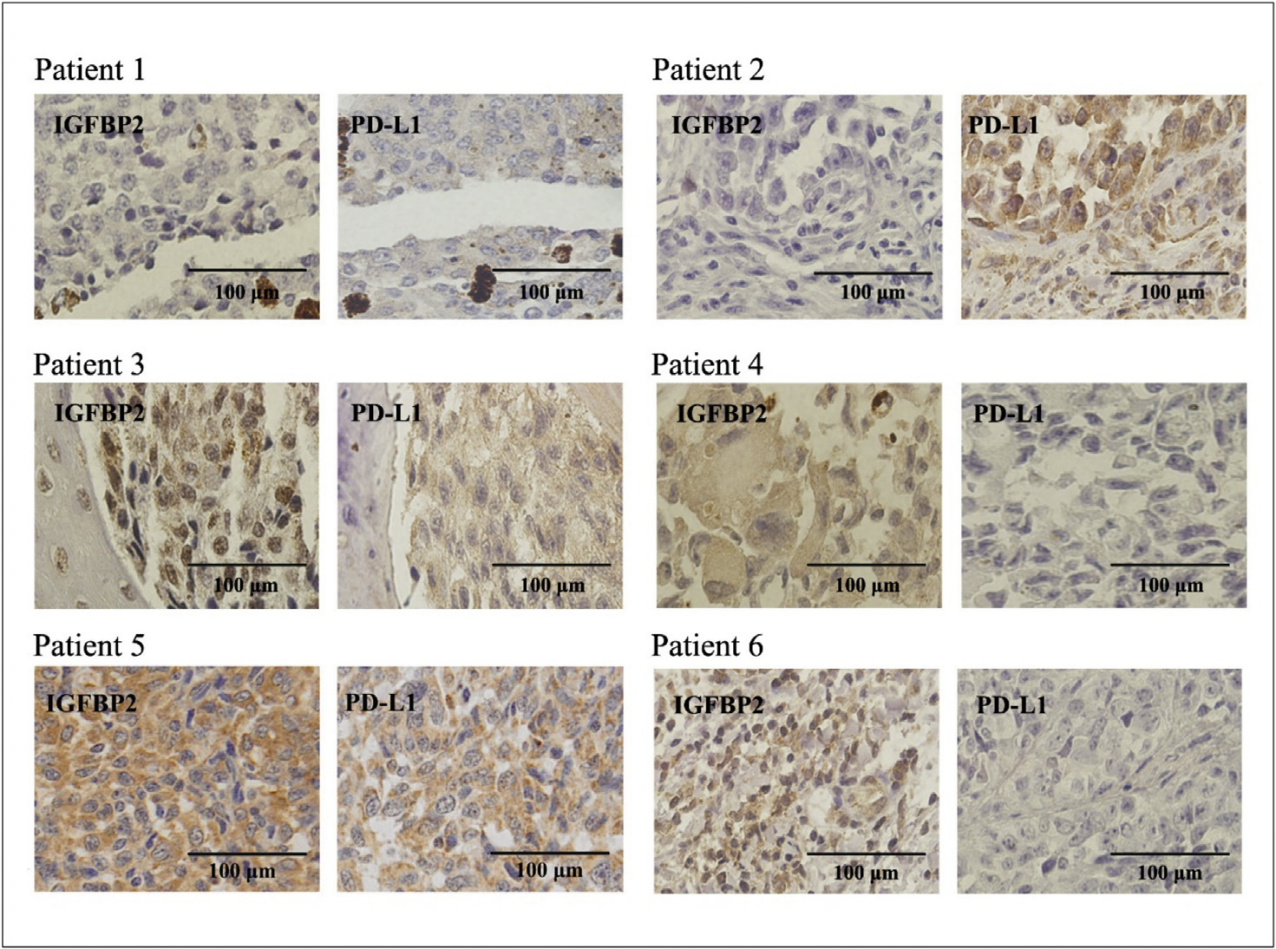
The IHC staining of 6 melanoma patients with IGFBP2 and PD-L1 expression. Patient 1 showed low IGFBP2 and low PD-L1 expression; Patient 2 showed low IGFBP2 and high PD-L1 expression; Patient 3 showed high IGFBP2 and high PD-L1 expression; Patient 4 showed high IGFBP2 and low PD-L1 expression; Patient 5 showed high IGFBP2 and high PD-L1 expression; Patient 6 showed high IGFBP2 and low PD-L1 expression.

These data show the expression characteristics of malignant melanoma patients with or without anti-PD-1 treatment at RNA and protein levels. As for melanoma patients who did not receive anti-PD-1 treatment, data were collected from Tianjin Medical University Cancer Institute & Hospital from February 1981 to May 2013. The multivariate analysis data are shown in Table 1. The RNA sequencing data from 28 melanoma patients receiving anti-PD-1 therapy were obtained from the GEO database (GSE78220) (Supplementary Information 1). According to the response to anti-PD-1 treatment, patients were divided into two groups: response and non-response group. Bioinformatic analysis are shown in Fig. 1. The ROC analysis of the data in Table 2 corresponds to Fig 1D. The clinical characteristics of 13 Chinese melanoma patients who received anti-PD-1 treatment are shown Table 3. The efficacy evaluation and protein expression characteristics of anti-PD-1 treatment were shown in Figs. 2 and 3. Furthermore, Fig. 2 and Table 3 are related, and Fig. 3 is representing an IHC summary on subset of patients that have been listed in Table 2.

**Table 1:** Multivariate analysis of the prognostic values of IGFBP2 and PD-L1 protein expression in malignant melanoma patients.

Parameter	Overall survival		
	HR	95% CI	P
Both high	2.512	1.206–5.232	0.014
IGFBP2 high +PD-L1 low	1.338	0.476–3.762	0.581
IGFBP2 low +PD-L1 high	1.487	0.062–3.337	0.336
Both low	–	–	–

**Table 2:** The ROC analysis the response for IGFBP2, PD-L1 and TWO—HIGH groups to anti-PD-1 treatment.

Variables	AUC	95% CI	Cut-off	Sensitivity (%)	Specificity (%)
IGFBP2	0.536	34.4–72.8	1.50	53.8	53.3
PD-L1	0.536	34.4–72.8	1.50	53.8	53.3
TWO—HIGH	0.667	54.3–79.0	1.50	100	33.3

**Table 3:** Clinical characteristics of 13 Chinese melanoma patients in stage IV who received anti-PD-1 treatment.

Patient	Sex	Age	Tumor site	Metastasis site	PD-1 antibody	Cycles	Efficacy	PD-L1 expression	IGFBP2 expression
1	Female	57	Mucous	Lymph node	Opdivo	2	SD	Low	Low
2	Male	64	Mucous	Left adrenal gland	Keytruda	4	PD	High	Low
3	Male	61	Derma	Right lung	Keytruda	2	SD	High	High
4	Female	42	Derma	Right adrenal gland	Keytruda	5	PD	Low	High
5	Male	60	Derma	Right lung	Keytruda	4	PR	High	High
6	Female	57	undetermined origin	Right subaxillary	Keytruda	2	PD	Low	High
7	Male	53	Derma	Lymph node	Opdivo	2	SD	–	–
8	Female	53	Mucous	Lymph node	Keytruda	3	–	–	–
9	Female	76	Derma	Left lung	Keytruda	21	–		–
10	Female	59	Mucous	Lymph node	Keytruda	4	–	–	–
11	Female	50	Mucous	Liver	Opdivo	8	–	–	–
12	Female	57	Mucous	Lung	Keytruda	2	–	–	–
13	Male	62	Derma	Liver	Keytruda	7	–	–	–

Abbreviations: PR, partial response; SD, stable disease; PD, progression disease.

## 2 Experimental design, materials, and methods

### 2.1 Bioinformatic analysis of RNA sequencing data of melanoma patients with anti-PD-1 therapy (GSE78220)

Analysis of RNA sequencing data from the GEO database (GSE78220), which includes 28 patients with malignant melanoma who received anti-PD-1 treatment [1]. According to the response to anti-PD-L1 treatment, patients were divided into two groups: response and non-response groups. Cluster analysis of RNA expression was performed using R (package pheatmap). The difference in the mRNA expression of PD-L1 was analyzed by the Boxplot (R ggplot2 package). According to the median mRNA levels of IGFBP2 and PD-L1, the 28 patients were divided into four groups (high IGFBP2+high PD-L1, high IGFBP2+low PD-L1, low IGFBP2+high PD-L1 and low IGFBP2+low PD-L1). Differences among the four groups were analyzed by Fisher exact test. *p<0.05, **p<0.01, and ***p<0.001.

### 2.2 Anti-PD-1 treatment efficacy and assessment

Data were collected from 13 melanoma patients who had failed prior chemotherapy and treated in the Tianjin Medical University Cancer Institute & Hospital between July 2015 and December 2018. These patients had unresectable stage III or IV malignant melanoma. The therapeutic dose of Keytruda (pembrolizumab) was 2 mg/kg, once every three weeks, and the therapeutic dose of Opdivo (nivolumab) was 3 mg/kg, once every two weeks. The cancer immunotherapy response was captured by Response Evaluation Criteria in Solid Tumors (RECIST) [2,3]. Tumors were reduced by at least 30% for more than 4 weeks is considered a partial response (PR). The maximum diameter of the target lesion increased by at least 20% or new lesions were identified as disease progression (PD). The sum of the maximum diameter of the target lesion reduced to less than PR or increased to less than PD was considered stable disease (SD).

## Appendix Supplementary materials

Image, application 1
